# Situs inversus in cardiothoracic surgery and its advances: a case report

**DOI:** 10.1093/jscr/rjaf213

**Published:** 2025-04-14

**Authors:** Rachel Hinds

**Affiliations:** Cardiothoracic Department, Sir Charles Gardiner Hospital, Hospital Avenue, Nedlands, Perth, WA 2034, Australia

**Keywords:** cardiothoracic, surgery, situs inversus, anatomy, transposition, anomaly

## Abstract

Situs inversus is a rare congenital abnormality, rarely encountered by surgeons. Despite its sparsity, knowledge and preparation for surgery in these individuals is imperative. In the field of cardiothoracic surgery new technology and techniques have offered new avenues to avoid complications. I will detail a case of a 73-year-old gentleman who attended for treatment of lung cancer with a background of situ inversus.

## Introduction

Situs inversus is a rare congenital abnormality—a mirror-image transposition of the thoracic abdominal organs. Situs inversus totalis (SIT) indicates the heart is also transposed—named dextrocardia. This condition falls under the heterotaxy or an abnormal left/right distribution of organs [1].

Situs inversus was first described in animals by Aristotle and later a reversal of liver and spleen was reported in humans in 1600 by Fabricius [2]. It affects ~1:1000 and favours men at a ratio of 1.5:1 [3]. Cardiac and vascular anomalies are increased in this population. It is asymptomatic but is absolutely crucial in diagnosis and surgical intervention. Imaging is important to help a surgeon plan their approach. In this report, I will detail the case of a 73-year-old gentleman male with SIT and lung cancer.

## Case

**Figure 1:**
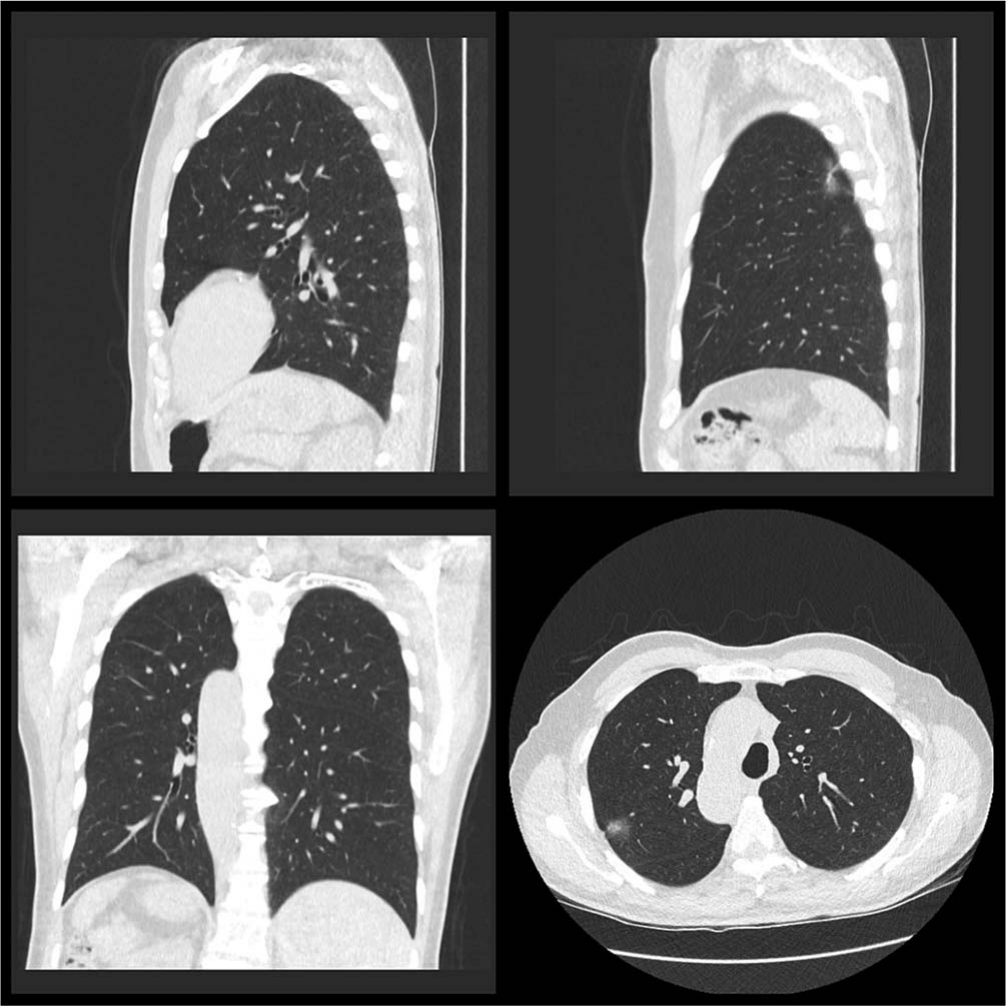
Pre-operative CT thorax, abdomen, pelvis.

A 73-year-old gentleman was referred to our cardiothoracic department with a tumour of his right upper and right lower lobe diagnosed on computed tomography (CT), as seen in Fig. 1. He initially presented to his general practitioner (GP) with fatigue and weight loss. His background included ex-smoker with five pack-year history having quit 40 years prior, mild chronic obstructive pulmonary disease type 2 diabetes mellitus, dyslipidaemia, peptic ulcer disease, gastro-oesophageal reflux disease, diverticulosis, and colonic polyps. The patient has been known to have SIT since his early adulthood, an incidental finding on a chest X-ray.

A low-dose CT chest (Fig. 1) showed a nodule in the right upper lobe of 20 × 20 mm in diameter with a central solid component 7 × 4 mm and an 8-mm diameter ground glass opacity with a central solid dot in the right lower lobe. No lymphadenopathy was detected.

He was referred for a CT-guided biopsy of the right upper lobe. This was followed by the standard positron emission tomography (PET) scan, which showed mild flurodeoxyglucose (FDG) uptake in the solid component of the nodule and some calcification in his mediastinal lymph nodules with no obvious nodal or distant metastasis. Given a diagnosis of lung adenocarcinoma stage 1a (cT1cN0M0), he was referred to cardiothoracic surgery for consideration of lobectomy.

He was for a video-assisted thoracic surgery (VATS); specifically right upper lobectomy/trigsegmentectomy +/− right lower lobe nodule wedge resection +/− segementectomy.

He underwent VATS right upper lobectomy and right lower lobe superior segmentectomy. Double-lumen intubation was employed for single lung ventilation. On inspection intraoperatively the oblique fissure of the right lung was 50% developed with morphological upper, middle and lower lobe, therefore confirming our impression it was a conventional right lung with incomplete fissure in the right chest space rather than a left lung in the right space as with usual situs inversus patients. A conventional right upper lobectomy was first performed and then attention turned to the right lower lobe nodule. The incomplete fissure made dissection of the right lower lobe apical segmental artery and bronchus challenging however, a wedge resection of the lower lobe nodule was sent for frozen section along with several lymph nodes, confirming adenocarcinoma with the absence of lymph node involvement; and he therefore progressed to a lower lobe superior segmentectomy with the view to preserve residual lung function.

**Figure 2:**
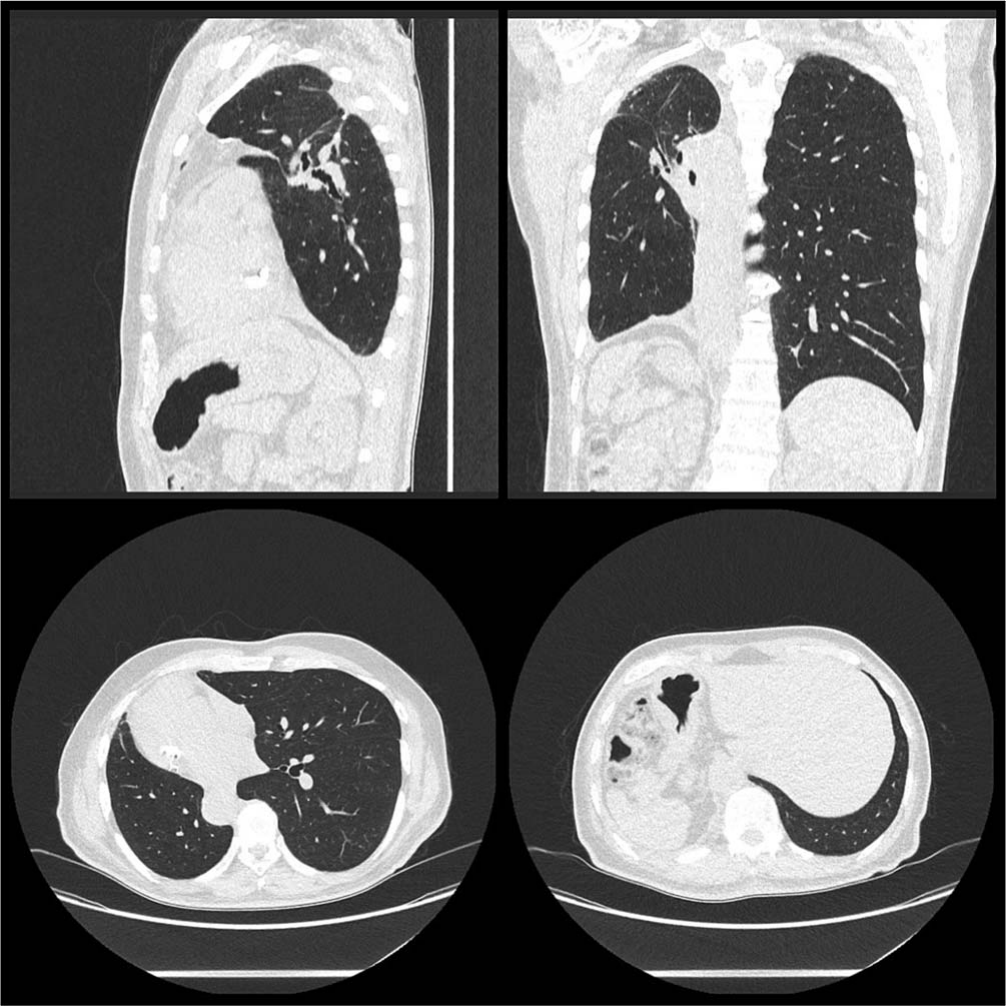
Post-operative CT thorax, abdomen, pelvis.

He was discharged post-operative Day 9. Histology confirmed moderately differentiated adenocarcinoma pT1cN0 in the right upper lobe and moderately differentiated adenocarcinoma pT1aN0 in the right lower lobe with clear margins for both. He was reviewed 2 weeks post-discharge and planned for surveillance with his respiratory physician (Fig. 2).

## Discussion

Situs inversus is believed to start with an abnormal rotation of the cardiac tube in the embryonic period due to disturbance of ciliary motility; hence, the link to Kartagener syndrome in 20%–25% of cases, a triad of situs inversus, chronic sinusitis, and bronchiectasis [4, 5]. Our case did not fall into this group. This rarity was first described by Aristotle in animals, later “The Father of Embryology” Fabricius reported a case of human liver and spleen reversal in 1600 [2]. It has since been reported several times and has an estimated occurrence of 1:6500 and 1:25 000 [6]. This anomaly has implications for surgery; making it necessary to have an extensive understanding of the anatomy prior to surgery and beneficial that our case was elective. Whilst situs inversus may be detected upon physical examination, imaging confirms and aids a surgeon in navigating their operation. Initially as with our patient plain film X-ray will often show dextrocardia and left-placed liver. However, advanced modalities such as CT and MRI give greater detail. In this case it was sufficient for our patient to undergo routine imaging prior to thoracic surgery. It has been suggested that pre-operative CT angiography may help further prepare for intra-operative challenges [7]. The use of CT-generated three-dimensional images remains useful in the preparation phase. Some studies noted the use of advanced software (e.g. Mimics Medical) that generates images combining CT bronchography and angiography to reconstruct the anatomy to clearly highlight the target and anticipate any anatomical complications in the pre-operative period [8].

At the time of surgery care needs to be taken to detect anatomical variants; notably the vascular variation, the relationship between pulmonary artery, vein, and bronchus can be altered in situs inversus. The path of the recurrent laryngeal nerve must be assessed due to its course along the aortic arch [9]. In our patient, it was noted that the oblique fissures were only 50% developed, making dissection difficult.

It presents issues for the anaesthetist; as usual in thoracic surgery, one lung ventilation is essential. However, all equipment would be designed for the more common rotation—i.e. a double-lumen tracheal tube would be designed to fit the left bronchus (anatomically the right in a patient with SIT). Our case used video-assisted thoracic surgery; the surgeon’s position may need to be altered in a case with SIT. A recent study reported the first use of robotic-assisted thoracic surgery, which would further increase the precision and signals a very exciting age to be in thoracic surgery [10].

## Conclusion

SIT can pose challenges for surgeons; thankfully extensive planning can be executed with modern-day imaging. The future of surgeons like this looks promising with the arrival of model-generating software and robotic surgery techniques. Though these are not widely available a lot can be anticipated from standard CT imaging and bronchoscopy; these patients can expect to do as well as those without SIT.

## Declaration

This case report was written and images used with permission from the patient; the patient has reviewed the finished draft and granted permission to publish. All patient identifiers have been omitted.
